# Bis(imidazole-κ*N* ^3^)bis­(nitrato-κ*O*)zinc(II)

**DOI:** 10.1107/S1600536809037672

**Published:** 2009-09-26

**Authors:** Adama Sy, Aliou Hamady Barry, Fatma Ben Amor, Ahmed Driss, Mohamed Gaye, Abdou Salam Sall

**Affiliations:** a Département de Chimie, Faculté des Sciences et Techniques, Université Cheikh Anta Diop, Dakar, Senegal; b Département de Chimie, Faculté des Sciences, Université de Nouakchott, Nouakchott, Mauritania; c Campus Universitaire, Département de Chimie, Faculté des Sciences, Université de Tunis, 1060 Tunis, Tunisia

## Abstract

The title complex, [Zn(NO_3_)_2_(C_3_H_4_N_2_)_2_], contains a Zn^II^ centre with a slightly distorted tetra­hedral coordination environment, involving two N atoms from imidazole ligands and two O atoms from nitrate anions. The imino NH groups participate in inter­molecular N—H⋯O hydrogen bonds.

## Related literature

For related structures, see: Li *et al.* (2007 ▶); Xie *et al.* (2009 ▶); He *et al.* (2007 ▶); Shaw *et al.* (2009 ▶).
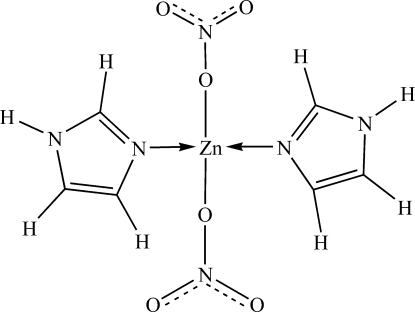


## Experimental

### 

#### Crystal data

[Zn(NO_3_)_2_(C_3_H_4_N_2_)_2_]
*M* *_r_* = 325.55
Triclinic, 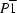

*a* = 7.785 (6) Å
*b* = 8.126 (2) Å
*c* = 11.394 (2) Å
α = 92.36 (2)°
β = 99.67 (4)°
γ = 96.32 (7)°
*V* = 704.9 (6) Å^3^
*Z* = 2
Mo *K*α radiation
μ = 1.77 mm^−1^
*T* = 293 K
0.1 × 0.1 × 0.1 mm

#### Data collection

Enraf–Nonius CAD-4 diffractometer
Absorption correction: none
3798 measured reflections
3068 independent reflections
2733 reflections with *I* > 2σ(*I*)
*R*_int_ = 0.014

#### Refinement

*R*[*F* ^2^ > 2σ(*F* ^2^)] = 0.044
*wR*(*F* ^2^) = 0.127
*S* = 1.07
3068 reflections
173 parameters
H-atom parameters not refined
Δρ_max_ = 0.53 e Å^−3^
Δρ_min_ = −0.64 e Å^−3^

### 

Data collection: *CAD-4 EXPRESS* (Enraf–Nonius, 1994 ▶); cell refinement: *CAD-4 EXPRESS*; data reduction: *CAD-4 EXPRESS*; program(s) used to solve structure: *SHELXS97* (Sheldrick, 2008 ▶); program(s) used to refine structure: *SHELXL97* (Sheldrick, 2008 ▶); molecular graphics: *ORTEP-3 for Windows* (Farrugia, 1997 ▶); software used to prepare material for publication: *SHELXL97*.

## Supplementary Material

Crystal structure: contains datablocks I, global. DOI: 10.1107/S1600536809037672/fj2244sup1.cif

Structure factors: contains datablocks I. DOI: 10.1107/S1600536809037672/fj2244Isup2.hkl

Additional supplementary materials:  crystallographic information; 3D view; checkCIF report

## Figures and Tables

**Table:** 

Zn1—O4	1.966 (3)
Zn1—O1	1.999 (3)
Zn1—N3	2.011 (3)
Zn1—N5	2.015 (3)

**Table:** 

O4—Zn1—O1	104.93 (12)
O4—Zn1—N3	113.61 (12)
O1—Zn1—N3	113.00 (11)
O4—Zn1—N5	95.75 (11)
O1—Zn1—N5	118.25 (12)
N3—Zn1—N5	110.03 (13)

**Table 2:** Hydrogen-bond geometry (Å, °)

*D*—H⋯*A*	*D*—H	H⋯*A*	*D*⋯*A*	*D*—H⋯*A*
N4—H4N⋯O1^i^	0.86	1.96	2.808 (4)	170
N6—H6N⋯O6^ii^	0.86	1.91	2.741 (4)	161

Symmetry codes: (i) 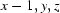; (ii) 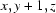.

**Fig. 1.**
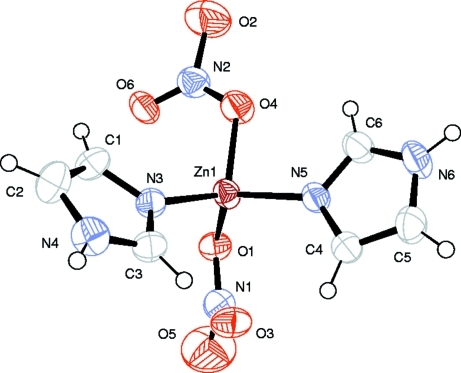
An ORTEP view of the asymmetric unit of the title compound, showing the atom-numbering scheme (for all no H-atoms). Displacement ellipsoids are plotted at the 50% probability level.

**Fig. 2.**
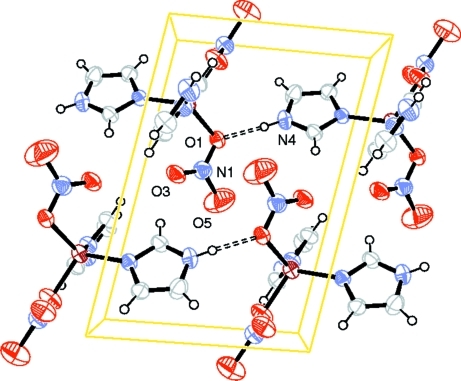
Molecular representation of the compound showing hydrogen bonds. The broken lines indicate hydrogen bonds.

## supplementary crystallographic information

### Comment

The asymmetric unit of the title compound, contains a Zn^II^ cation, two imidazole ligands and two nitrate anions acting as monodentate ligands (Fig. 1). In the molecule the Zn^II^ atom is four-coordinated in a distorted tetrahedral configuration by two N atoms from two imidazole molecules and two O atoms from monodentate two nitrate groups (Table 1). The angles O4—Zn—N5 and O1—Zn—O4 are reduced while all the others angles are increased in comparison with the ideal tetrahedral angle of 109.5° (Li *et al.*, 2007) The values of Zn–N distances, 2.011 (3) and 2.015 (3) Å, are little far to that found for tris(2-ethyl-1*H*-imidazole-κ*N*^3^)(terephthalato-κ*O*)zinc(II) (Xie *et al.* 2009) and bis(1*H*-imidazole-κ*N*^3^)[(2-oxidobenzylideneamino)methanesulfonato-κ^2^*N*,*O*]zinc(II) (He *et al.* 2007). The Zn—O coordinating distances of 1.966 (4) and 1.999 (3) Å are comparable of those found in diphenyldipyrazolylmethane complexes with zinc(II) (Shaw *et al.* 2009). The mononuclear complex is joined into a two-dimensional layer by N—H···O type hydrogen-bonds; details have been provided in Table 2.

### Experimental

Zinc(II) acetate dihydrate (0.1320 g; 0.6 mmol) and lanthanum nitrate hexahydrate (0.0433 g; 0.01 mmol) were dissolved in 10 ml of a mixture of water and methanol (1/2). To this solution was added imidazole (0.0408 g; 0.6 mmol) and tartaric acid (0.0900 g; 0.6 mmol) dissolved in 12 ml of an aqueous NaOH 0.1 *M* solution. After 120 m of stirring, a solution of tartaric acid (0.0900 g; 0.6 mmlol) in 5 ml of methanol was added again. The reaction mixture give white solid which was filtered and dried in air. The filtrate was left to crystallize. The crystals of (I) which formed were filtered off and dried [yield 82%]. Analysis calculated for [Zn(C_3_H_4_N_2_)_2_(NO_3_)_2_]: C 22.14, H 2.48, N 25.81%; found: C 22.09, H 2.46, N 25.78%. Spectroscopic analysis, IR (ν, cm^-1^): 3111, 3058, 1621, 1603, 1571, 1543, 1449, 1332 and 1072. The IR spectra were recorded with a Nicolet Magna 760 IR spectrophotometer in KBr pellets.

### Refinement

All H atoms were placed geometrically and refined with a riding model. *U*_iso_(H) for H was assigned as 1.2*U*_eq_ of the attached C atoms.

### Crystal data

**Table:**

[Zn(NO_3_)_2_(C_3_H_4_N_2_)_2_]	*Z* = 2
*M**_r_* = 325.55	*F*(000) = 328
Triclinic, *P*1	*D*_x_ = 1.534 Mg m^−^^3^
Hall symbol: -P 1	Mo *K*α radiation, λ = 0.71073 Å
*a* = 7.785 (6) Å	Cell parameters from 25 reflections
*b* = 8.126 (2) Å	θ = 11–15°
*c* = 11.394 (2) Å	µ = 1.77 mm^−^^1^
α = 92.36 (2)°	*T* = 293 K
β = 99.67 (4)°	Prism, colourless
γ = 96.32 (7)°	0.1 × 0.1 × 0.1 mm
*V* = 704.9 (6) Å^3^	

### Data collection

**Table:**

Enraf–Nonius CAD-4 diffractometer	2733 reflections with *I* > 2σ(*I*)
Radiation source: fine-focus sealed tube	*R*_int_ = 0.014
graphite	θ_max_ = 27.0°, θ_min_ = 2.5°
ω scans	*h* = −9→2
3798 measured reflections	*k* = −10→10
3068 independent reflections	*l* = −14→14

### Refinement

**Table:**

Refinement on *F*^2^	Secondary atom site location: difference Fourier map
Least-squares matrix: full	Hydrogen site location: inferred from neighbouring sites
*R*[*F*^2^ > 2σ(*F*^2^)] = 0.044	H-atom parameters not refined
*wR*(*F*^2^) = 0.127	*w* = 1/[σ^2^(*F*_o_^2^) + (0.0746*P*)^2^ + 0.6727*P*] where *P* = (*F*_o_^2^ + 2*F*_c_^2^)/3
*S* = 1.07	(Δ/σ)_max_ = 0.003
3068 reflections	Δρ_max_ = 0.53 e Å^−^^3^
173 parameters	Δρ_min_ = −0.64 e Å^−^^3^
0 restraints	Extinction correction: *SHELXL97* (Sheldrick, 2008), Fc^*^=kFc[1+0.001xFc^2^λ^3^/sin(2θ)]^-1/4^
Primary atom site location: structure-invariant direct methods	Extinction coefficient: 0.017 (3)

### Special details

**Table:**

Geometry. All e.s.d.'s (except the e.s.d. in the dihedral angle between two l.s. planes) are estimated using the full covariance matrix. The cell e.s.d.'s are taken into account individually in the estimation of e.s.d.'s in distances, angles and torsion angles; correlations between e.s.d.'s in cell parameters are only used when they are defined by crystal symmetry. An approximate (isotropic) treatment of cell e.s.d.'s is used for estimating e.s.d.'s involving l.s. planes.
Refinement. Refinement of *F*^2^ against ALL reflections. The weighted *R*-factor *wR* and goodness of fit *S* are based on *F*^2^, conventional *R*-factors *R* are based on *F*, with *F* set to zero for negative *F*^2^. The threshold expression of *F*^2^ > σ(*F*^2^) is used only for calculating *R*-factors(gt) *etc*. and is not relevant to the choice of reflections for refinement. *R*-factors based on *F*^2^ are statistically about twice as large as those based on *F*, and *R*- factors based on ALL data will be even larger.

### Fractional atomic coordinates and isotropic or equivalent isotropic displacement parameters (Å^2^)

**Table:**

	*x*	*y*	*z*	*U*_iso_*/*U*_eq_	
Zn1	0.15424 (5)	0.41605 (4)	0.23967 (3)	0.03654 (17)	
O1	0.3369 (3)	0.3103 (3)	0.3456 (2)	0.0430 (5)	
O2	0.3189 (6)	0.3208 (6)	−0.0920 (4)	0.0971 (13)	
O3	0.1851 (4)	0.4038 (4)	0.4754 (2)	0.0547 (6)	
O4	0.2364 (4)	0.4269 (3)	0.0859 (2)	0.0558 (7)	
O5	0.4232 (7)	0.2470 (7)	0.5506 (5)	0.1252 (18)	
O6	0.2253 (4)	0.1542 (3)	0.0596 (2)	0.0574 (7)	
N1	0.3052 (4)	0.3279 (4)	0.4537 (3)	0.0510 (7)	
N2	0.2542 (4)	0.2947 (4)	0.0259 (3)	0.0503 (7)	
N3	−0.0884 (3)	0.2952 (3)	0.2279 (2)	0.0360 (5)	
N4	−0.3596 (4)	0.2487 (4)	0.2566 (3)	0.0477 (7)	
H4N	−0.4528	0.2558	0.2868	0.057*	
N5	0.1481 (4)	0.6622 (3)	0.2647 (2)	0.0385 (6)	
N6	0.1656 (4)	0.9234 (3)	0.2214 (3)	0.0509 (7)	
H6N	0.1862	1.0114	0.1842	0.061*	
C1	−0.1781 (5)	0.1749 (4)	0.1439 (3)	0.0440 (7)	
H1	−0.1308	0.1226	0.0847	0.053*	
C2	−0.3456 (5)	0.1454 (5)	0.1615 (4)	0.0535 (9)	
H2	−0.4340	0.0700	0.1178	0.064*	
C3	−0.2038 (4)	0.3359 (4)	0.2941 (3)	0.0417 (7)	
H3	−0.1791	0.4147	0.3581	0.050*	
C4	0.0906 (5)	0.7536 (4)	0.3524 (3)	0.0429 (7)	
H4	0.0509	0.7110	0.4189	0.051*	
C5	0.1012 (5)	0.9154 (4)	0.3262 (4)	0.0507 (8)	
H5	0.0708	1.0031	0.3704	0.061*	
C6	0.1905 (5)	0.7699 (4)	0.1878 (3)	0.0445 (7)	
H6	0.2325	0.7422	0.1187	0.053*	

### Atomic displacement parameters (Å^2^)

**Table:**

	*U*^11^	*U*^22^	*U*^33^	*U*^12^	*U*^13^	*U*^23^
Zn1	0.0395 (2)	0.0308 (2)	0.0420 (2)	0.00520 (14)	0.01374 (15)	0.00335 (14)
O1	0.0397 (12)	0.0507 (13)	0.0420 (12)	0.0110 (10)	0.0144 (10)	0.0003 (10)
O2	0.124 (3)	0.106 (3)	0.069 (2)	0.009 (3)	0.041 (2)	0.013 (2)
O3	0.0595 (16)	0.0674 (17)	0.0446 (13)	0.0221 (13)	0.0207 (12)	0.0009 (12)
O4	0.0794 (19)	0.0442 (13)	0.0528 (15)	0.0118 (13)	0.0342 (14)	0.0027 (11)
O5	0.130 (4)	0.144 (5)	0.095 (3)	0.039 (3)	−0.018 (3)	0.028 (3)
O6	0.082 (2)	0.0396 (13)	0.0546 (15)	−0.0006 (13)	0.0282 (14)	0.0086 (11)
N1	0.0515 (17)	0.0519 (17)	0.0489 (16)	0.0010 (14)	0.0108 (13)	0.0008 (13)
N2	0.0495 (17)	0.0553 (18)	0.0472 (16)	0.0039 (14)	0.0126 (13)	0.0046 (13)
N3	0.0364 (13)	0.0365 (13)	0.0361 (13)	0.0051 (10)	0.0094 (10)	0.0005 (10)
N4	0.0363 (14)	0.0550 (17)	0.0559 (17)	0.0096 (12)	0.0167 (13)	0.0031 (14)
N5	0.0459 (14)	0.0319 (12)	0.0396 (13)	0.0048 (11)	0.0118 (11)	0.0051 (10)
N6	0.063 (2)	0.0332 (14)	0.0614 (19)	0.0060 (13)	0.0214 (16)	0.0147 (13)
C1	0.0432 (17)	0.0485 (18)	0.0397 (16)	0.0090 (14)	0.0062 (13)	−0.0084 (14)
C2	0.0428 (19)	0.052 (2)	0.061 (2)	0.0028 (15)	0.0023 (16)	−0.0091 (17)
C3	0.0446 (17)	0.0444 (17)	0.0385 (16)	0.0070 (14)	0.0141 (13)	−0.0018 (13)
C4	0.0528 (19)	0.0368 (16)	0.0407 (16)	0.0038 (14)	0.0138 (14)	0.0030 (13)
C5	0.063 (2)	0.0346 (16)	0.058 (2)	0.0075 (15)	0.0181 (18)	0.0001 (15)
C6	0.0516 (19)	0.0407 (17)	0.0453 (17)	0.0067 (14)	0.0178 (15)	0.0090 (13)

### Geometric parameters (Å, °)

**Table:**

Zn1—O4	1.966 (3)	N4—H4N	0.8600
Zn1—O1	1.999 (3)	N5—C6	1.320 (4)
Zn1—N3	2.011 (3)	N5—C4	1.383 (4)
Zn1—N5	2.015 (3)	N6—C6	1.334 (5)
O1—N1	1.301 (4)	N6—C5	1.372 (5)
O2—N2	1.526 (5)	N6—H6N	0.8600
O3—N1	1.228 (4)	C1—C2	1.350 (5)
O4—N2	1.282 (4)	C1—H1	0.9300
O5—N1	1.532 (5)	C2—H2	0.9300
O6—N2	1.229 (4)	C3—H3	0.9300
N3—C3	1.327 (4)	C4—C5	1.356 (5)
N3—C1	1.381 (4)	C4—H4	0.9300
N4—C3	1.330 (5)	C5—H5	0.9300
N4—C2	1.369 (5)	C6—H6	0.9300
			
O4—Zn1—O1	104.93 (12)	C4—N5—Zn1	131.1 (2)
O4—Zn1—N3	113.61 (12)	C6—N6—C5	107.5 (3)
O1—Zn1—N3	113.00 (11)	C6—N6—H6N	126.2
O4—Zn1—N5	95.75 (11)	C5—N6—H6N	126.2
O1—Zn1—N5	118.25 (12)	C2—C1—N3	109.0 (3)
N3—Zn1—N5	110.03 (13)	C2—C1—H1	125.5
N1—O1—Zn1	107.0 (2)	N3—C1—H1	125.5
N2—O4—Zn1	121.2 (2)	C1—C2—N4	106.4 (3)
O3—N1—O1	121.1 (3)	C1—C2—H2	126.8
O3—N1—O5	122.4 (4)	N4—C2—H2	126.8
O1—N1—O5	116.5 (3)	N3—C3—N4	110.7 (3)
O6—N2—O4	123.7 (3)	N3—C3—H3	124.6
O6—N2—O2	120.5 (3)	N4—C3—H3	124.6
O4—N2—O2	115.8 (3)	C5—C4—N5	109.2 (3)
C3—N3—C1	105.9 (3)	C5—C4—H4	125.4
C3—N3—Zn1	124.1 (2)	N5—C4—H4	125.4
C1—N3—Zn1	129.5 (2)	C4—C5—N6	106.2 (3)
C3—N4—C2	108.0 (3)	C4—C5—H5	126.9
C3—N4—H4N	126.0	N6—C5—H5	126.9
C2—N4—H4N	126.0	N5—C6—N6	111.5 (3)
C6—N5—C4	105.5 (3)	N5—C6—H6	124.2
C6—N5—Zn1	123.2 (2)	N6—C6—H6	124.2

### Hydrogen-bond geometry (Å, °)

**Table:**

*D*—H···*A*	*D*—H	H···*A*	*D*···*A*	*D*—H···*A*
N4—H4N···O1^i^	0.86	1.96	2.808 (4)	170
N6—H6N···O6^ii^	0.86	1.91	2.741 (4)	161

Symmetry codes: (i) *x*−1, *y*, *z*; (ii) *x*, *y*+1, *z*.
